# 2426. High Reliability Line Stewardship: A Novel Approach to Effective Central Line Associated Bloodstream Infection Risk Reduction

**DOI:** 10.1093/ofid/ofad500.2045

**Published:** 2023-11-27

**Authors:** Nancy Kerr, Jayme Bland, Karen Pasco, Sukrut Dwivedi, Rajan Gurunathan, Nicole Hughes, Christopher Moran, Alyssa Roman

**Affiliations:** Hackensack Meridian Ocean University Medical Center, Brick, New Jersey; Hackensack Meridian Health Ocean University Medical Center, Brick, New Jersey; Hackensack Meridian Health Ocean University Medical Center, Brick, New Jersey; Hackensack Meridian Ocean University Medical Center, Brick, New Jersey; Hackensack Meridian Health, Edison, New Jersey; Hackensack Meridian Health Ocean University Medical Center, Brick, New Jersey; Hackensack Meridian Health Ocean University Medical Center, Brick, New Jersey; Hackensack Meridian Health Ocean University Medical Center, Brick, New Jersey

## Abstract

**Background:**

Hospital-acquired infections (HAIs) impact health care quality and may cause patient harm. Central to the goal of preventable harm reduction are the tenets of high-reliability, specifically, the principle of deference to expertise and the principle of sensitivity to operations. Barriers can include lack of adherence to standardized criteria in support of best practice, inconsistent application of key concepts related to improvement and reliability, and limited time, resources and engagement.

We set forth to design and implement an effective and sustainable ‘line stewardship’ program, in hopes of improving utilization of CVCs and reducing central line associated bloodstream infection (CLABSI) risk at our academic community hospital in central New Jersey.

**Methods:**

Jan - Feb 2022 : Baseline outcomes measured

Mar - May 2022 : Assessment and planning

Jul - Dec 2022 : Intervention and tracking

Jan - Feb 2023 : Follow-up outcomes measured

Intervention Phases

**Figure:**
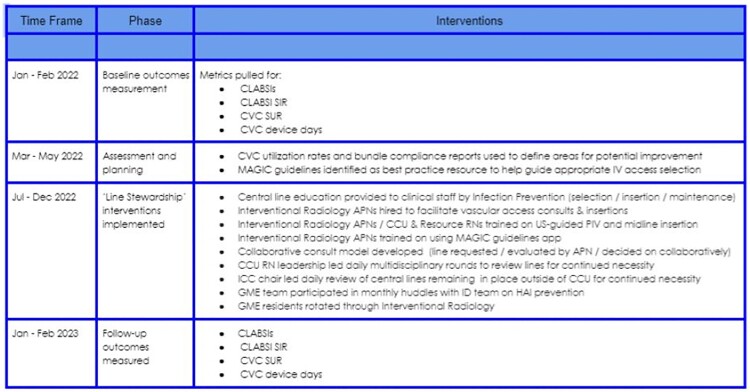

**Results:**

During the 6 mon. intervention and tracking phase, OUMC experienced a 37% reduction in CVC utilization, with a decrease in the CVC utilization ratio (SUR, as defined by NHSN, from .751 to .537), yielding a reduction of over 550 line days/mon. When compared with baseline, the observed / expected infection rate decreased thru intervention phase (2.365 to 0.347), with an associated decline of CLABSIs. In comparison to the Jan - Feb 2022 data period, results from Jan - Feb 2023 continued to trend favorably, with both a continued decline in SUR (0.448), and sustained use of CVC alternatives (23.5 devices/mon).

**Figure:**
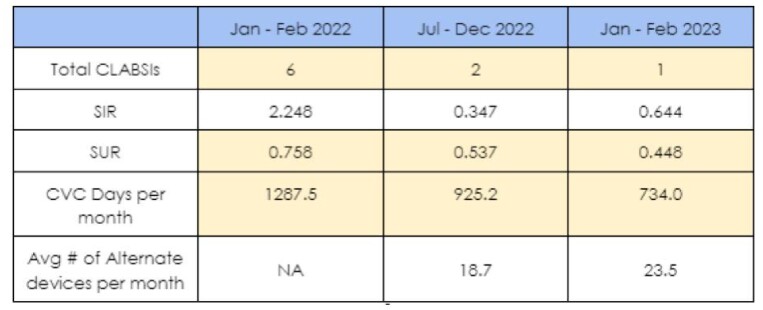

**Figure:**
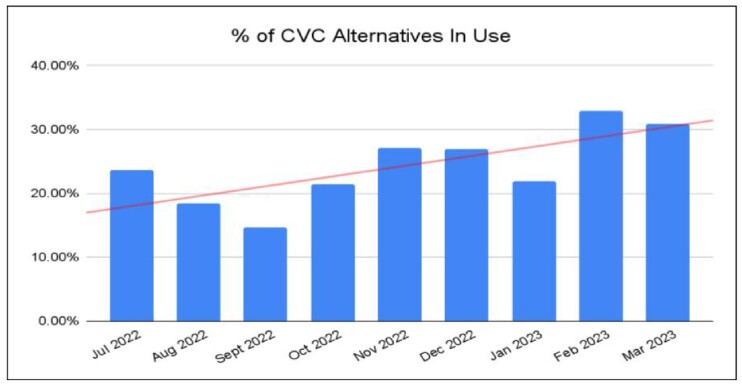

**Conclusion:**

The concept of stewardship is well-known in infectious disease circles and mirror principles of high-reliability and harm reduction, care standards, structured processes, and collaborative expertise are key to ensuring consistency and sustaining performance. After some initial push-back from providers who wanted to maintain their autonomy with access selection, the development of our vascular access order pathway and collaborative consult model became well-accepted as a way of triaging appropriate access to fit each patient. Our experience suggests that education, provider engagement, and a shared focus on care guidelines and reliability principles, can be the basis for a sustainable and effective CLABSI risk reduction strategy.

**Disclosures:**

**All Authors**: No reported disclosures

